# Supplementary material for: Structural basis for stabilisation of the RAD51 nucleoprotein filament by BRCA2
Source: Nat Commun. 2023 Nov 2;14:7003. doi: 10.1038/s41467-023-42830-1 (PMC10622577; doi:10.1038/s41467-023-42830-1)
Supplement: Supplementary file 1 — Supplementary information [file 41467_2023_42830_MOESM1_ESM.pdf]

Supplementary table 1. DNA oligos and peptides

Oligos	Size (nt)	Sequence (5' – 3')
**Mutagenesis**		
D184A		TATGGTCTCTCTGGCAGTGCTGTCCTGGATAATGTAGCCTATGC (forward), CCTCTCAGCCACTGCCAGCAGCCGTTC (reverse)
D184A, D187A		TCTGGCAGTGCTGTCCTGGCTAATGTAGCCTATGCTCGCG (forward), GAGACCATACCTCTCAGCCACTGCCAGCAG (reverse)
**EMSA, SPR, CryoEM (micrographs, 2D classification)**		
**Figure 1**		
Panels C, D (ssDNA)	60	**FAM**-ATGGTGTGTGTAGGTTAATGTGAGGAGGAGAGGTGAAGAAGGAGGAGAGAAGAAGGAGGC
Panels C, D (dsDNA)	60	**FAM**-ATGGTGTGTGTAGGTTAATGTGAGGAGGAGAGGTGAAGAAGGAGGAGAGAAGAAGGAGGC, GCCTCCTTCTTCTCTCCTCCTTCTTCACCTCTCCTCCTCACATTAACCTACACACACCAT
Panels E, F (ssDNA)	60	**Biotin**-ATGGTGTGTGTAGGTTAATGTGAGGAGGAGAGGTGAAGAAGGAGGAGAGAAGAAGGAGGC
Panels E, F (dsDNA)	60	**Biotin**-ATGGTGTGTGTAGGTTAATGTGAGGAGGAGAGGTGAAGAAGGAGGAGAGAAGAAGGAGGC, GCCTCCTTCTTCTCTCCTCCTTCTTCACCTCTCCTCCTCACATTAACCTACACACACCAT
**Related to Figure 1:**		
Supplementary figs. 3, 4 (ssDNA)	60	**Biotin**-ATGGTGTGTGTAGGTTAATGTGAGGAGGAGAGGTGAAGAAGGAGGAGAGAAGAAGGAGGC
Supplementary figs. 3, 4 (dsDNA)	60	**Biotin**-ATGGTGTGTGTAGGTTAATGTGAGGAGGAGAGGTGAAGAAGGAGGAGAGAAGAAGGAGGC, GCCTCCTTCTTCTCTCCTCCTTCTTCACCTCTCCTCCTCACATTAACCTACACACACCAT
**Figure 2**		
Panel A (ssDNA)	32	**Cy5**-TTTTTTTTTTTCGTGTGGTACTTTTTTTTTTT
Panel B (dsDNA)	50	CCGACTGACGCTCAACATAGGTACCACACGGCGAGCTCGATGCACCTCCA-**Cy3**, TGGAGGTGCATCGAGCTCGCGACAAACCTTCTATGTTGAGCGTCAGTCGG
Panel B (ssDNA)	60	**Biotin**-ATGGTGTGTGTAGGTTAATGTGAGGAGGAGAGGTGAAGAAGGAGGAGAGAAGAAGGAGGC-**Biotin**
Panel B (dsDNA)	60	**Biotin**-ATGGTGTGTGTAGGTTAATGTGAGGAGGAGAGGTGAAGAAGGAGGAGAGAAGAAGGAGGC-**Biotin**, GCCTCCTTCTTCTCTCCTCCTTCTTCACCTCTCCTCCTCACATTAACCTACACACACCAT
Panel C (ssDNA)	32	**Biotin**-TTTTTTTTTTTCGTGTGGTACTTTTTTTTTTT-**Biotin**
Panel C (dsDNA)	50	**Biotin**-CCGACTGACGCTCAACATAGGTACCACACGGCGAGCTCGATGCACCTCCA-**Cy3**, **Biotin**-TGGAGGTGCATCGAGCTCGCGACAAACCTTCTATGTTGAGCGTCAGTCGG
**Related to Figure 2:**		
Supplementary fig. 5A	50	CCGACTGACGCTCAACATAGGTACCACACGGCGAGCTCGATGCACCTCCA-**Cy3**, TGGAGGTGCATCGAGCTCGCGACAAACCTTCTATGTTGAGCGTCAGTCGG
Supplementary fig. 5B (ssDNA)	32	**Cy5**-TTTTTTTTTTTCGTGTGGTACTTTTTTTTTTT-**Biotin**,
Supplementary fig. 5B (ssDNA)	32	**Biotin**-TTTTTTTTTTTCGTGTGGTACTTTTTTTTTTT-**Biotin**
Supplementary fig. 5B (dsDNA)	50	**Biotin**-TGGAGGTGCATCGAGCTCGCGACAAACCTTCTATGTTGAGCGTCAGTCGG, **Biotin**-CCGACTGACGCTCAACATAGGTACCACACGGCGAGCTCGATGCACCTCCA-**Cy3**
**Figure 4**		
Panels B, C, D (ssDNA)	60	**FAM**-ATGGTGTGTGTAGGTTAATGTGAGGAGGAGAGGTGAAGAAGGAGGAGAGAAGAAGGAGGC
Panels F, G (ssDNA)	60	GCCTCCTTCTTCTCTCCTCCTTCTTCACCTCTCCTCCTCACATTAACCTACACACACCAT
**CryoEM (3D)**		
RAD51-ssDNA-TR2	60	**Biotin**-ATGGTGTGTGTAGGTTAATGTGAGGAGGAGAGGTGAAGAAGGAGGAGAGAAGAAGGAGGC-**Biotin**
RAD51-dsDNA-TR2	50	**Biotin**-CCGACTGACGCTCAACATAGGTACCACACGGCGAGCTCGATGCACCTCCA-**Cy3**, **Biotin**-TGGAGGTGCATCGAGCTCGCGACAAACCTTCTATGTTGAGCGTCAGTCGG

Peptides	Size (aa)	Sequence
TR2	49	DDQKNCKKRRALDFLSRLPLPPPVSPICTFVSPAAQKAFQPPRSCGTKY (BRCA2 residues 3260 to 3308, Genosphere)
Cy3-TR2	49	**Cy3**-DDQKNCKKRRALDFLSRLPLPPPVSPICTFVSPAAQKAFQPPRSCGTKY (BRCA2 residues 3260 to 3308, Genosphere)
BRC4	35	EKIKEPTLLGFHTASGKKVKIAKESLDKVKNLFDE (BRCA2 residues 1514 to 1548; Merck)

FAM: 6-carboxyfluorescein; Cy3: cyanine 3; Cy5: cyanine 5.

Supplementary Table 2. CryoEM data collection and real-space refinement

	RAD51 - ssDNA - TR2		RAD51 - dsDNA - TR2	
Data Collection				
Microscope	Titan Krios G3		Titan Krios G3	
Voltage (keV)	300		300	
Detector	K3		K3	
Collection mode	Counting		Counting	
Magnification	130,000x		130,000x	
Defocus range (mm)	-2.5 to -0.9 (0.2)		-2.5 to -0.9 (0.2)	
Num. movies	12,005		10,167	
Frames/movie	38		46	
Pixel size (Å/pixel)	0.652		0.652	
Electron dose ($e^-/Å^2/s$)	52.60		50.67	
Exposure (s)	1.04		1.25	
Picked particles	1,795,678		1,435,103	
Final particles	333,393		147,350	
Map resolution (Å):	masked	unmasked	masked	unmasked
d_{FSC} half maps, 0.143	2.93	3.11	3.26	3.43
d_{FSC} model, 0.143	2.62	2.62	2.83	2.86
Helical twist (°)	56.2		56.0	
Helical rise (Å)	16.1		15.8	
Real-space refinement				
Composition:				
Non-H atoms	28447		26366	
Residues:				
Protein	3581		3245	
Nucleotide	30		54	
Water	-		-	
Ligands:				
ATP	11		10	
Ca^{2+}	22		20	
Correlation coefficients[1]:				
CC, mask	0.80		083	
CC, peaks	0.66		0.58	
CC, volume	0.76		0.79	
<CC>, ligands	0.84		0.87	
Bonds (rmsd):				
Length(Å)	0.007		0.007	
Angles (°)	0.526		0.519	
MolProbity score[2]	1.97		1.92	
Clash score	7.21		5.21	
Ramachandran plot (%):				
Outliers	0.00		0.31	
Allowed	2.50		6.23	
Favoured	97.50		93.46	
Rotamer outliers (%)	4.22		1.92	
ADP (B-factors), mean:				
Protein	31.19		52.32	
Nucleotide	182.25		116.69	
Ligand	12.67		42.22	
Water	-		-	

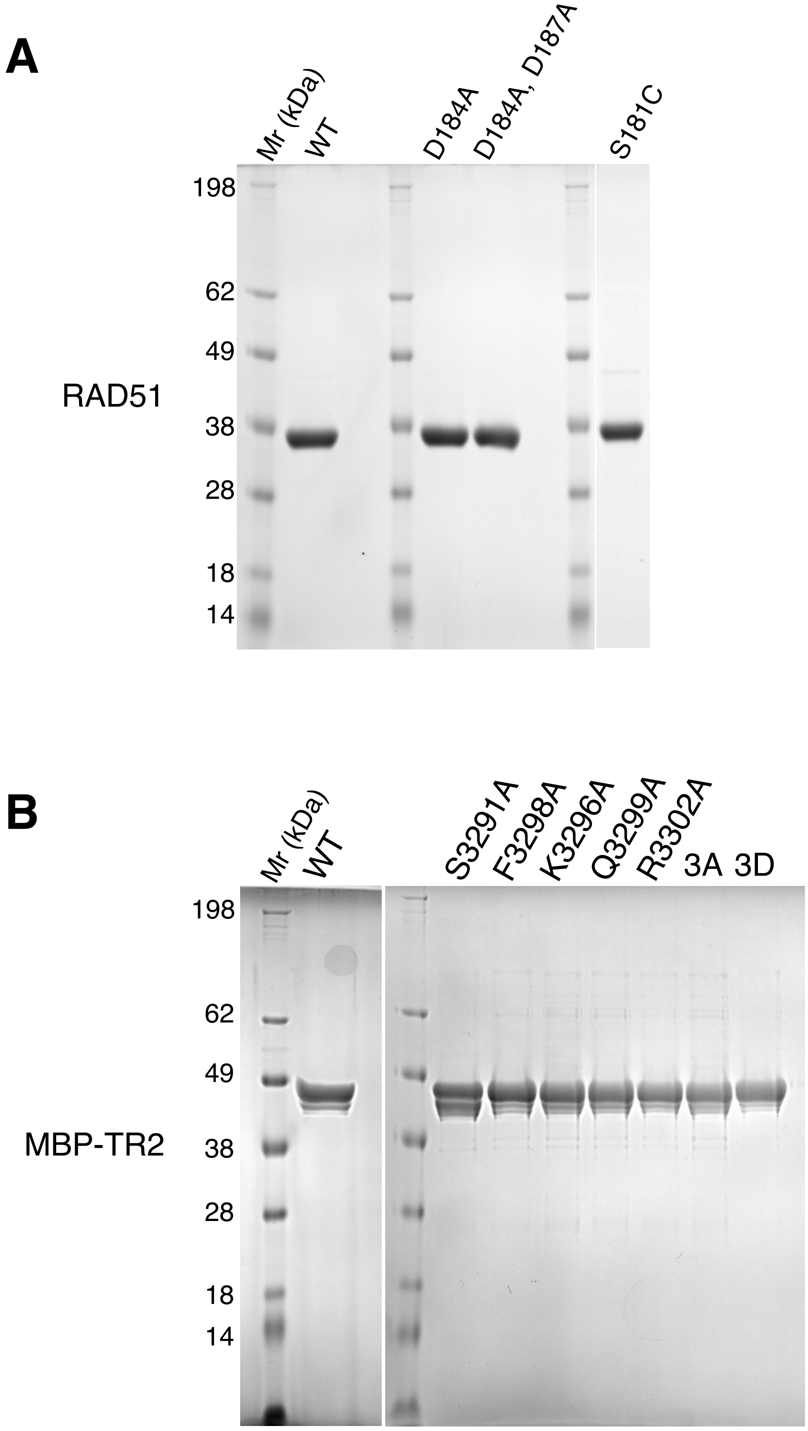

Supplementary figure 1. SDS-PAGE of purified RAD51 (A) and MBP-TR2 (B) proteins used in this study.

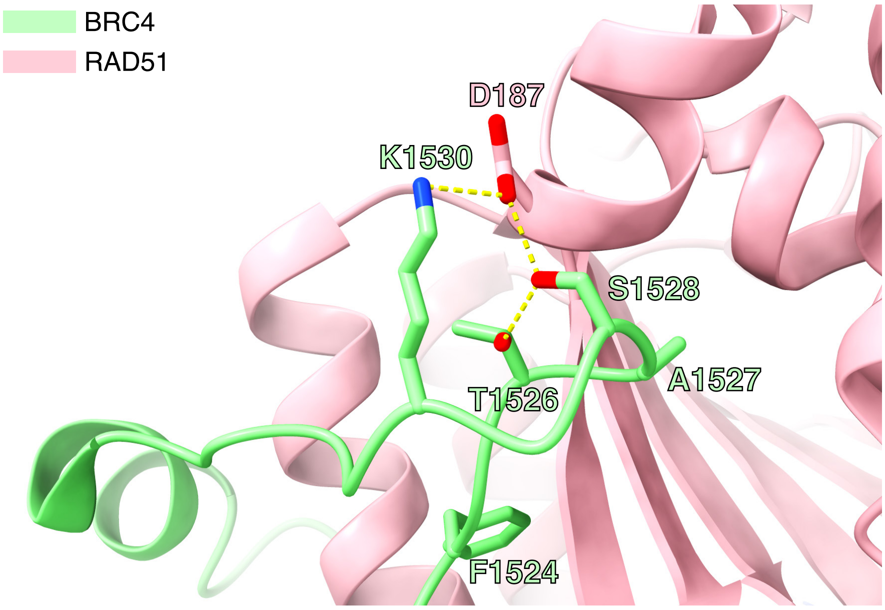

Supplementary figure 2. RAD51 D187 mediates BRCA2 BRC4 binding. Crystal structure of the RAD51 - BRC4 complex (PDB ID: 1N0W). The side-chain carboxylate of D187 supports a network of hydrogen bonds with the side chains of BRC4 hairpin loop residues T1526, S1528 and K1530.

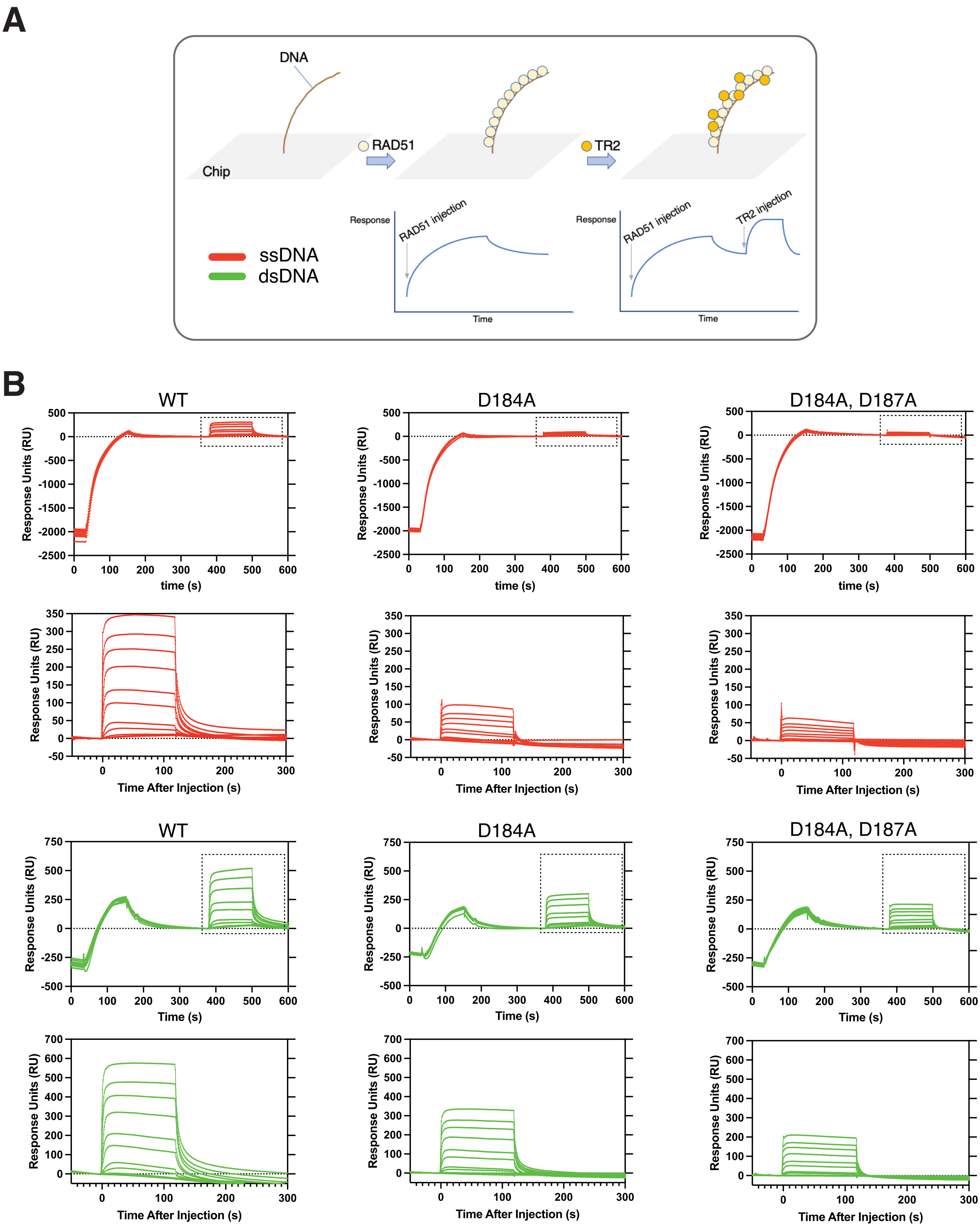

Supplementary figure 3. SPR analysis of the interaction of BRCA2 TR2 with the RAD51 NPF. **A** Schematic drawing of the SPR experiment. **B** Sensorgrams for TR2 binding to NPFs of wild-type, D184A and D184A, D187A RAD51 proteins and ss- (red traces, top) or dsDNA (green traces, bottom). For both ss- and dsDNA, the top row shows the full extent of the sensorgram for wild-type and mutant RAD51 proteins and the bottom row shows an expanded view of the sensorgram region corresponding to injection of the peptide (dashed box).

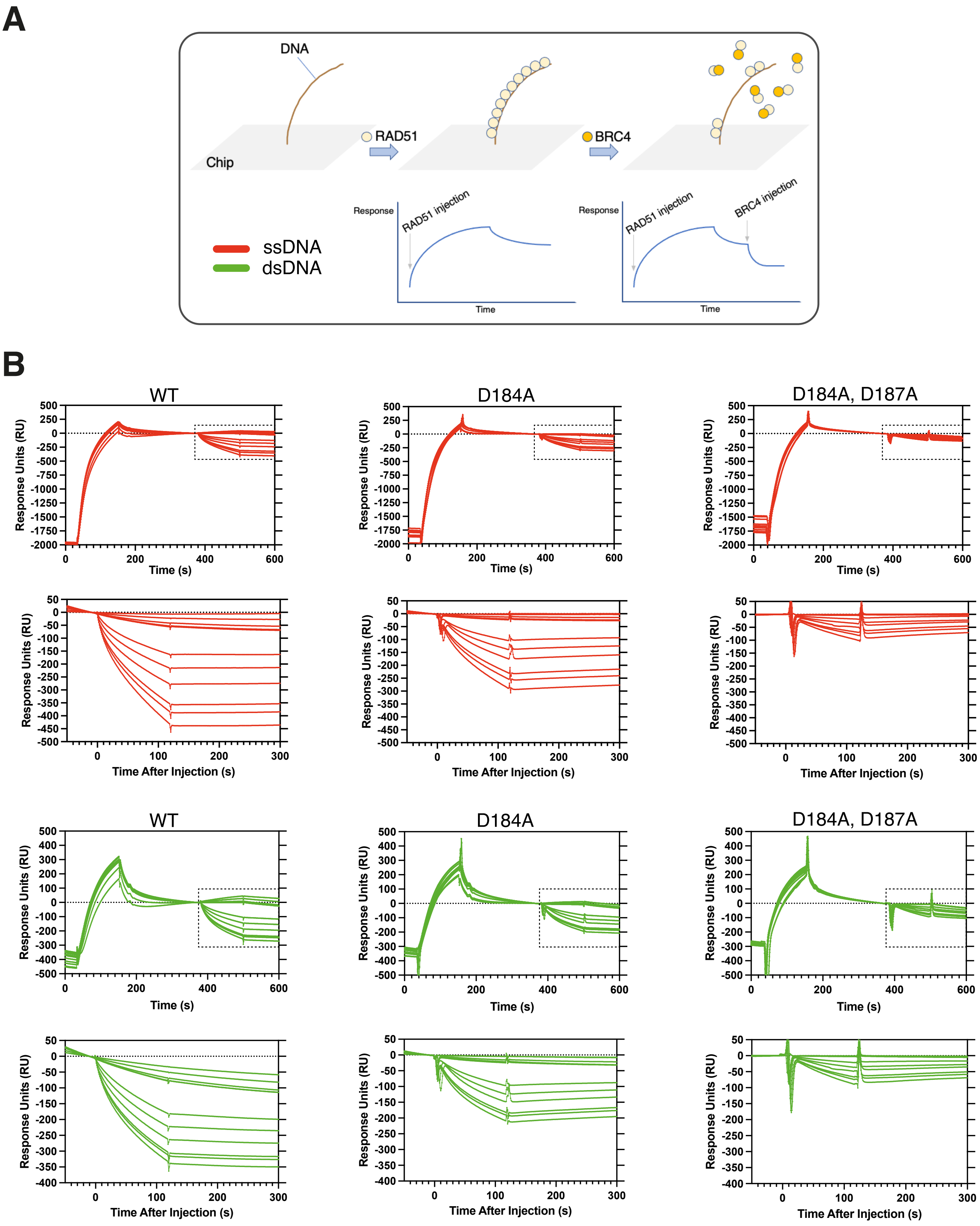

Supplementary figure 4. SPR analysis of the interaction of BRCA2 BRC4 with the RAD51 NPF. **A** Schematic drawing of the SPR experiment. **B** Sensorgrams for BRC4 binding to NPFs of wild-type, D184A and D184A, D187A RAD51 proteins and ss- (red traces, top) or dsDNA (green traces, bottom). For both ss- and dsDNA, the top row shows the full extent of the sensorgram for wild-type and mutant RAD51 proteins and the bottom row shows an expanded view of the sensorgram region corresponding to injection of the peptide (dashed box).

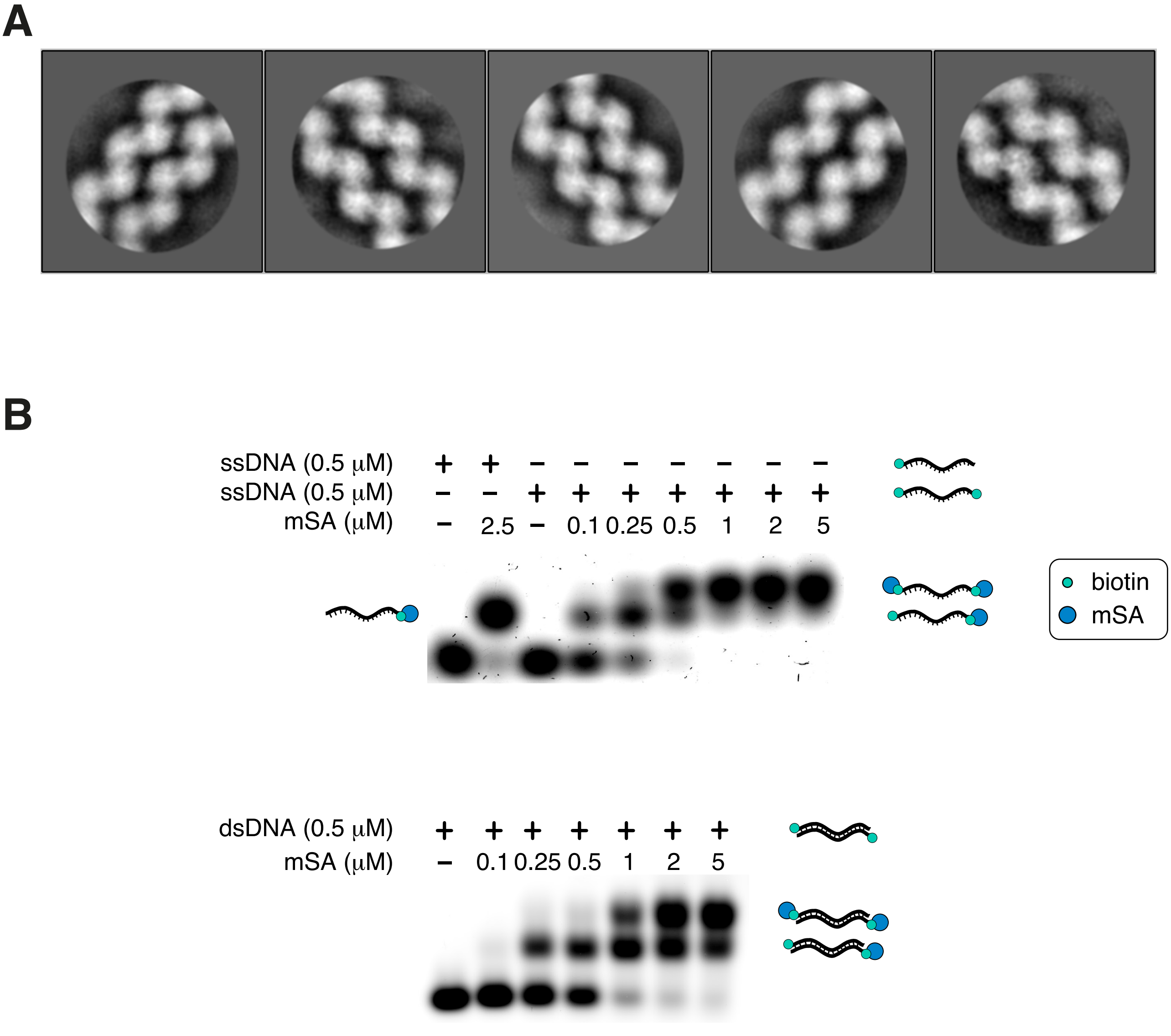

Supplementary figure 5. RAD51 filament bundling by BRCA TR2. A 2D classes showing pairs of RAD51 NPFs, obtained from particle alignment of bundled RAD51 - dsDNA NPFs in the presence of TR2. **B** EMSA analysis of mono-streptavidin (mSA) binding to singly- and doubly-biotinylated ssDNA (top) and doubly-biotinylated dsDNA (bottom). DNA was visualised by SYBR gold staining (ssDNA) or Cy3-tag fluorescence (dsDNA). Both experiments performed once.

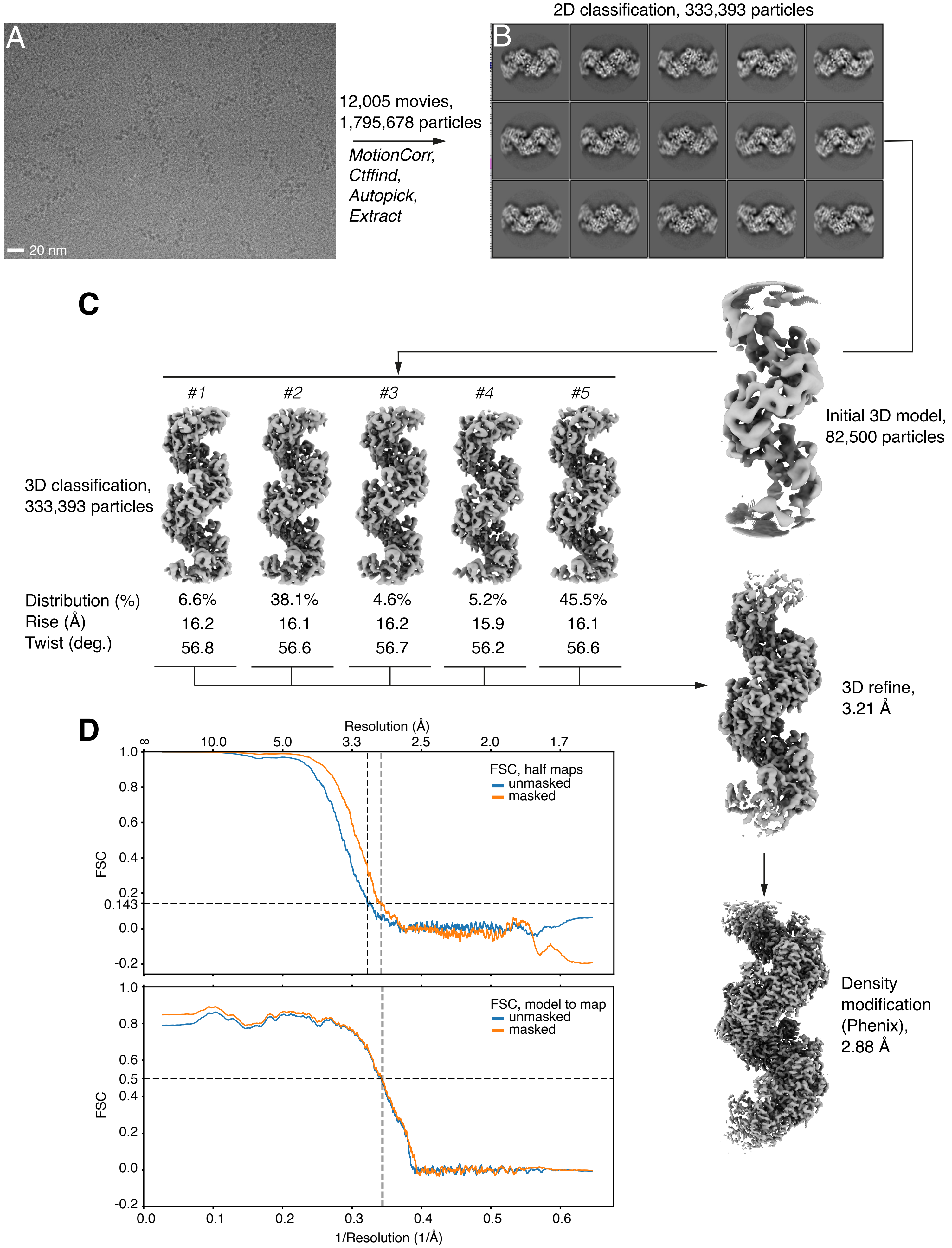

Supplementary figure 6. CryoEM data processing and reconstruction of the RAD51 - ssDNA - TR2 filament. A Representative micrograph. **B** Representative 2D classes. **C** 3D classification and refinement. **D** Half maps (top) and model-to-map (bottom) Fouries Shell Correlation curves.

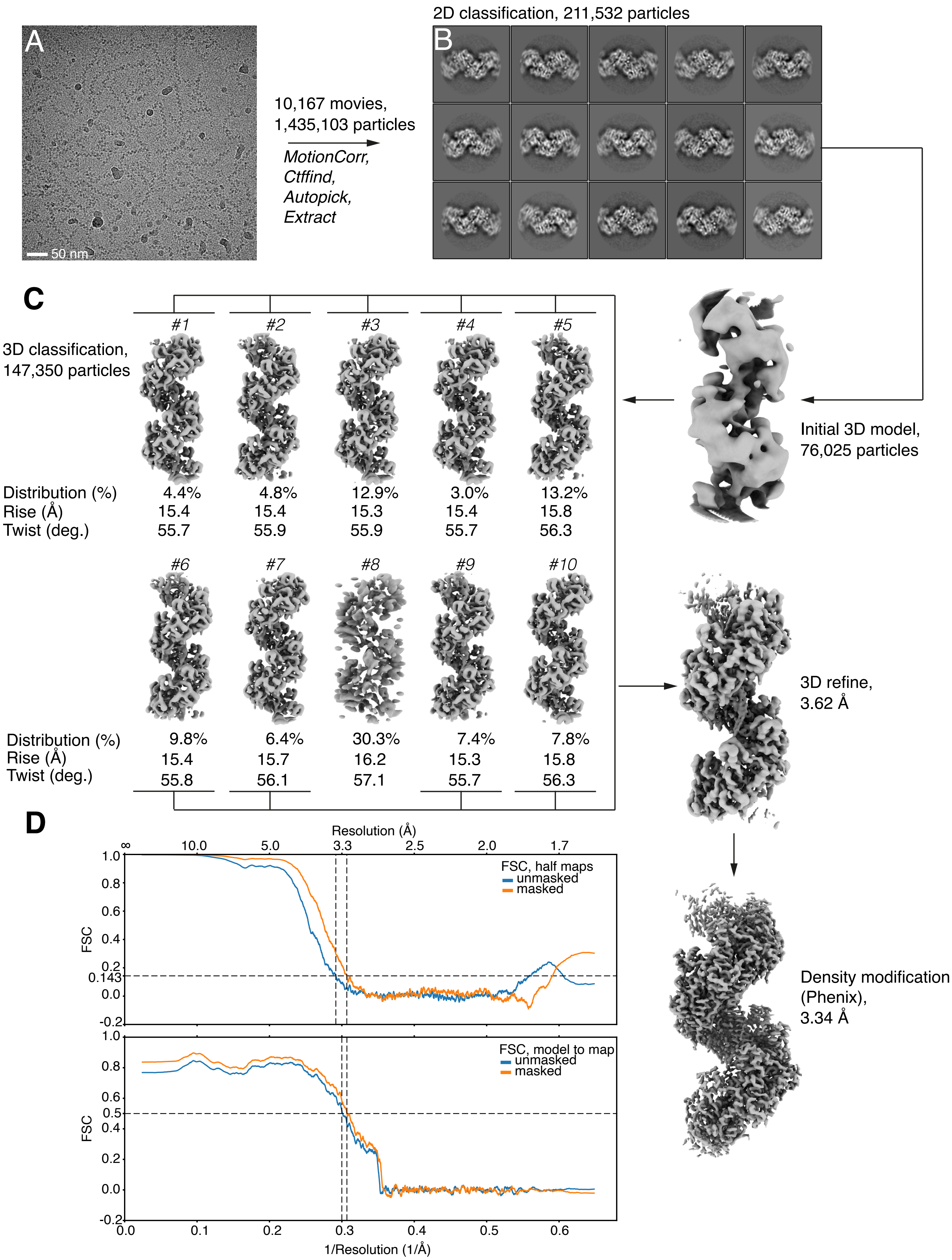

Supplementary figure 7. CryoEM data processing and reconstruction of the RAD51 - dsDNA - TR2 filament. A Representative micrograph. **B** Representative 2D classes. **C** 3D classification and refinement. **D** Half maps (top) and model-to-map (bottom) Fouries Shell. Correlation curves.

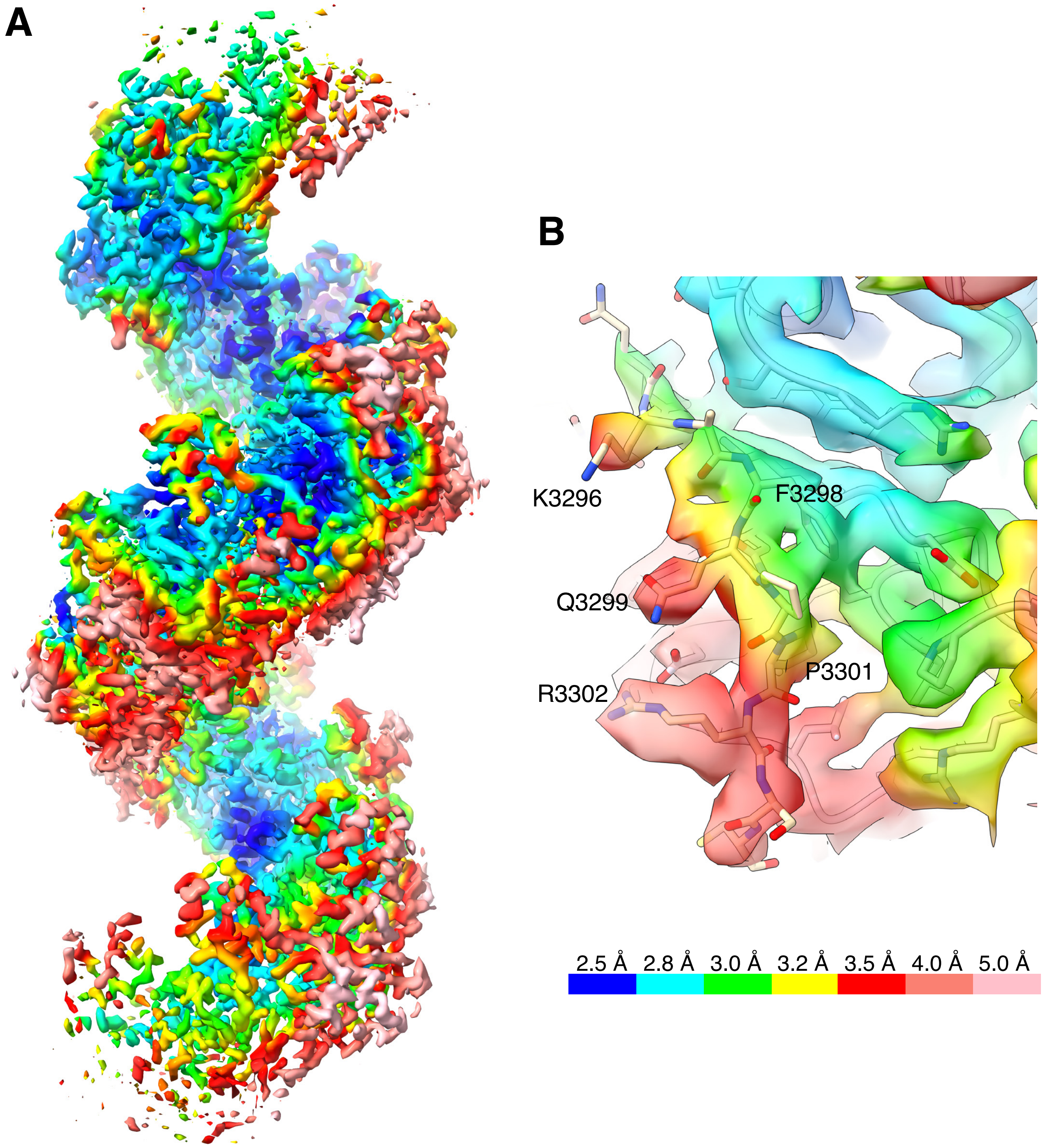

Supplementary figure 8. CryoEM map of the RAD51 - ssDNA - TR2 filament coloured according to local resolution. A Overall view of the map. B Closeup view of the map at the TR2 site. The colour scheme for the local resolution (2.5 Å to 5.0 Å) is shown under the panel.

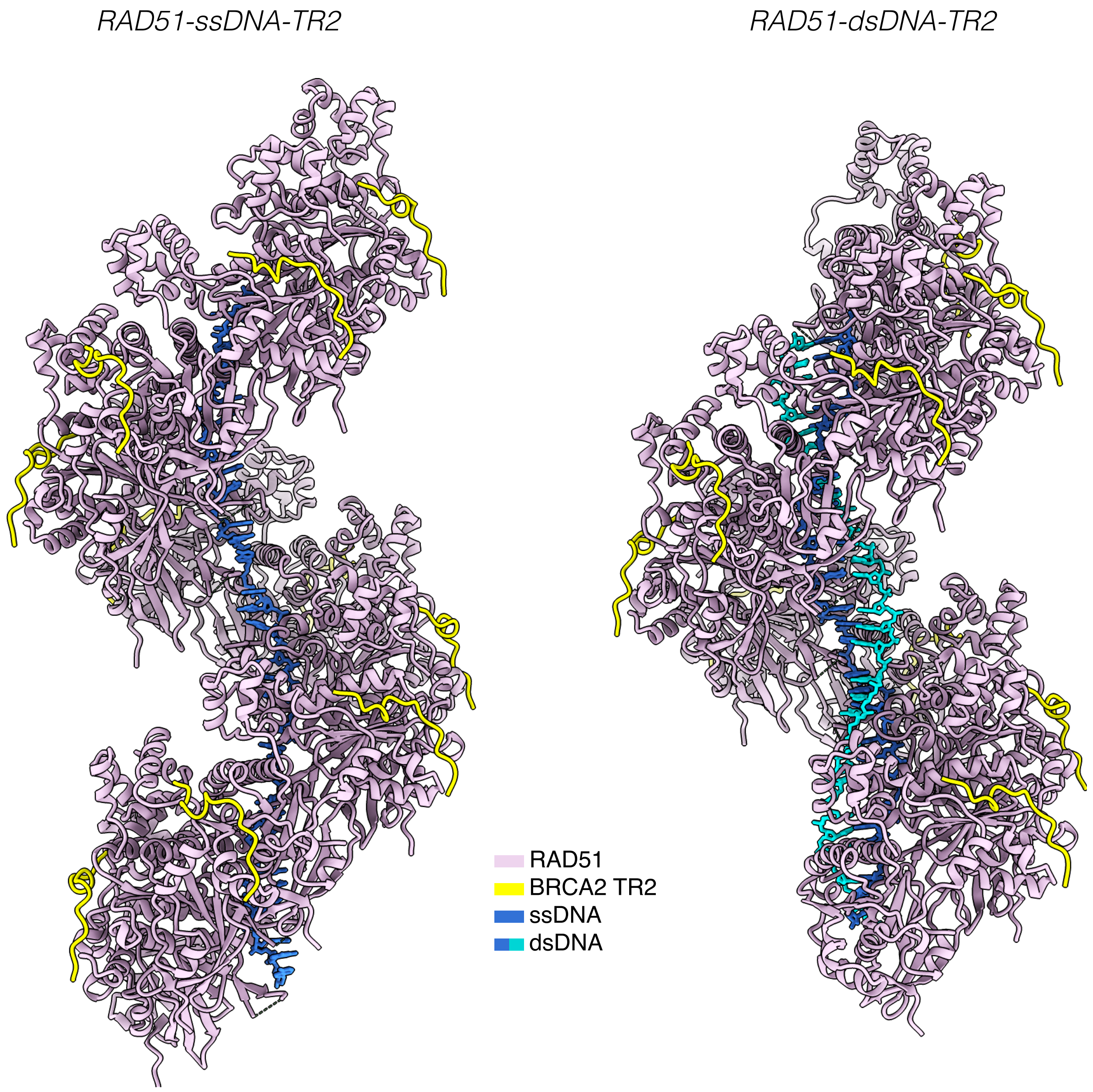

Supplementary figure 9. Side-by-side comparison of the structure of RAD51 - TR2 filaments on ss- and dsDNA.

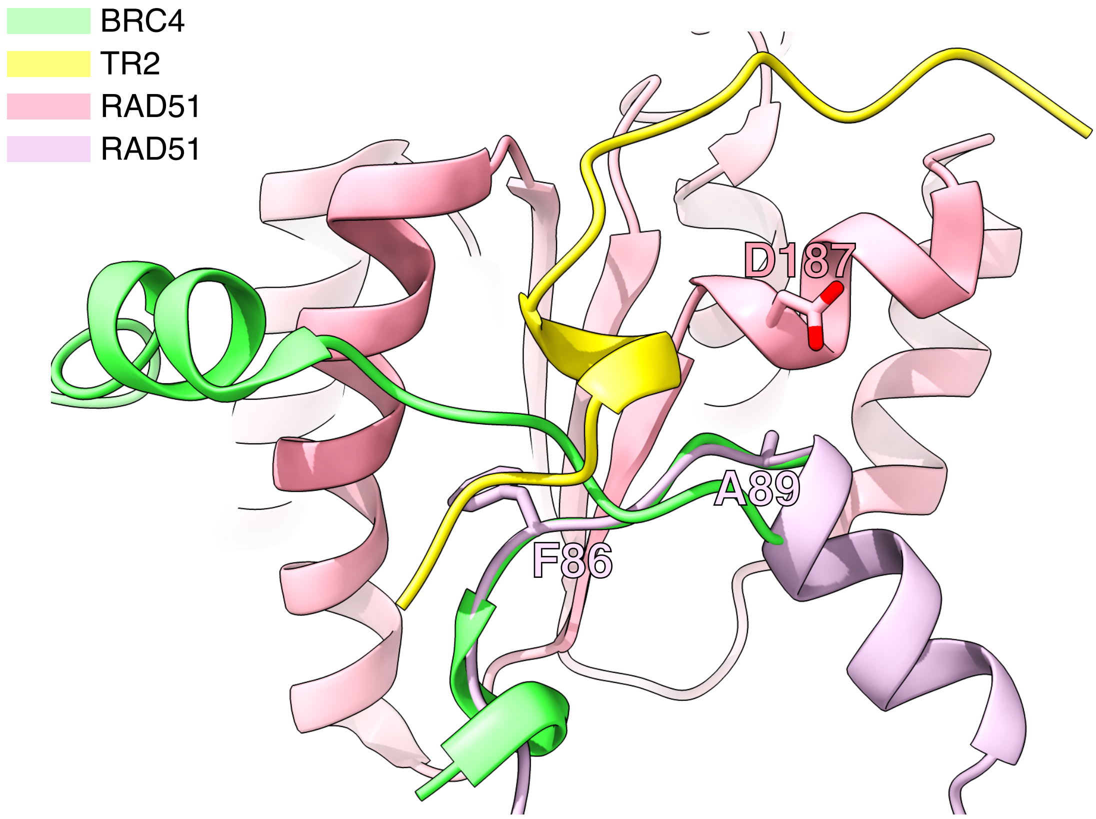

Supplementary figure 10. Overlap of the BRCA2 BRC4 and TR2 binding sites on the RAD51 surface. Superposition of RAD51 structures with bound BRC4 (PDB ID: 1N0W) and TR2 (this work). The side chains of residues F86, A89 and D187 in the adjacent RAD51.

Supplementary References:

1. Afonine PV *et al.* New tools for the analysis and validation of cryo-EM maps and atomic models. Acta Crystallographica, Section D: Structural Biology. 2018;74(9):814–840.

2. Chen VB *et al.*, MolProbity: all-atom structure validation for macromolecular crystallography. Acta Crystallographica Section D: Biological Crystallography. 2010;66(Pt 1):12–2
